# Focal Impulse and Rotor Modulation Ablation vs. Pulmonary Vein isolation for the treatment of paroxysmal Atrial Fibrillation: results from the FIRMAP AF study

**DOI:** 10.1093/europace/euaa378

**Published:** 2020-12-22

**Authors:** Roland R Tilz, Corinna Lenz, Philipp Sommer, Meyer-Saraei Roza, Anne E Sarver, Christopher G Williams, Christian Heeger, Gerhard Hindricks, Julia Vogler, Charlotte Eitel

**Affiliations:** 1 University Heart Center Lübeck, Medical Clinic II, Department of Electrophysiology, Lübeck, Germany; 2 Unfallkrankenhaus Berlin, Klinik für Innere Medizin/Kardiologie, Berlin, Germany; 3 Heart Center Leipzig, Department of Electrophysiology, Leipzig, Germany; 4 Abbott, Plymouth, MN, USA

**Keywords:** Atrial fibrillation, Catheter ablation, Rotor, Focal Impulse and Rotor Modulation

## Abstract

**Aims:**

Pulmonary vein isolation (PVI) is the gold standard for atrial fibrillation (AF) ablation. Recently, catheter ablation targeting rotors or focal sources has been developed for treatment of AF. This study sought to compare the safety and effectiveness of Focal Impulse and Rotor Modulation (FIRM)-guided ablation as the sole ablative strategy with PVI in patients with paroxysmal AF.

**Methods and results:**

We conducted a multicentre, randomized trial to determine whether FIRM-guided radiofrequency ablation without PVI (FIRM group) was non-inferior to PVI (PVI group) for treatment of paroxysmal AF. The two primary efficacy end points were (i) acute success defined as elimination of AF rotors (FIRM group) or isolation of all pulmonary veins (PVI group) and (ii) long-term success defined as single-procedure freedom from AF/atrial tachycardia (AT) recurrence 12 months after ablation. The study was closed early by the sponsor. At the time of study closure, any pending follow-up visits were waived. A total of 51 patients (mean age 63 ± 10.6 years, 57% male) were enrolled. All PVs were successfully isolated in the PVI group and all rotors were successfully eliminated in the FIRM group. Single-procedure effectiveness was 31.3% (5/16) in the FIRM group and 80% (8/10) in the PVI group at 12 months. Three vascular access complications occurred in the FIRM group.

**Conclusion:**

These partial study effectiveness results reinforce the importance of PVI in paroxysmal AF patients and indicate that FIRM-guided ablation alone (without PVI) is not an effective strategy for treatment of paroxysmal AF in most patients.

**What’s new?**

- To the best of our knowledge, the FIRMAP AF trial was the first randomized trial assessing the safety and efficacy of rotor ablation as the sole ablative strategy [without pulmonary vein isolation (PVI)] compared with the gold standard of conventional PVI in patients with paroxysmal atrial fibrillation (AF).
- Elimination of all rotors in the FIRM group and isolation of all pulmonary veins in the PVI group could be achieved in all patients.
- Single-procedure effectiveness was very poor in the FIRM group as compared with the PVI group at 6 months (52.9% vs. 85.7%) and 12 months (31.3% vs. 80%), respectively.
- Repeat catheter ablation procedures were performed more frequently in the FIRM group than in the PVI group (45.8% vs. 7.4%).
- The results suggest that FIRM-guided ablation alone (without PVI) is not an effective strategy for treatment of paroxysmal AF in most patients and reinforce the importance of PVI in paroxysmal AF patients.

## Introduction

Based on the assumption of trigger elimination, pulmonary vein isolation (PVI) currently presents the gold standard of atrial fibrillation (AF) ablation. Recently, rapidly spinning rotors or very rapid focal impulse formation have been raised as crucial sustaining mechanisms of AF.^1^ Catheter ablation at these relatively circumscribed areas has been shown to significantly affect AF, either by substantial slowing of the rate or termination to an atrial tachycardia (AT) or sinus rhythm.^2^ Targeted ablation of these sources, called Focal Impulse and Rotor Modulation (FIRM)-guided procedures, potentially obviates the need for trigger elimination with PVI. The RhythmView™ (Abbott, Menlo Park, CA, USA) system for Focal Impulse and Rotor Mapping (FIRM) analyses clinical AF electrograms to identify drivers. Ablation of these sites has shown variable success.^3–7^ The CONFIRM trial compared PVI with FIRM ablation followed by conventional PVI in patients with paroxysmal and mostly persistent AF.^8^ An on-treatment analysis of this trial suggested that success of conventional PVI depends solely on the coincidental ablation of underlying stable AF sources.^1^ In line with this hypothesis, the multicentre PRECISE trial demonstrated that FIRM-guided ablation only in patient-specific bi-atrial locations without concomitant PVI was associated with high success rates in 31 patients with paroxysmal AF (freedom from AF in 82.6% of patients after 190 days).^9^ However, publication of these data is still pending.

Until now there has not been a randomized study comparing FIRM ablation only with conventional PVI only in patients with paroxysmal AF. The aim of this study was to compare the safety and effectiveness of FIRM-guided catheter ablation only with the gold standard of PVI in patients with paroxysmal AF.

## Methods

### Recruitment and study design

This is an investigator initiated, prospective, multicentre, single-blinded, randomized study. Randomization was performed in subjects meeting the inclusion criteria (e.g. age 18–80 years, at least two documented episodes of paroxysmal AF in the 3 months preceding study entry, indication for AF ablation) and none of the exclusion criteria after signature of the study informed consent form. Block randomization stratified by centre was used to assign subjects (1:1) to the conventional radiofrequency (RF) ablation treatment with PVI (PVI group), or to the FIRM-guided RF ablation procedure without PVI (FIRM group). Additional ablation of any AT and/or the cavotricuspid isthmus (CTI) was allowed in those subjects with documented AT or typical atrial flutter.

The subjects were blinded to study treatment for the duration of the study period. Due to the nature of the study, it was not feasible to blind treating physicians.

The study was approved by the local ethics committees and registered under NCT02703454 by Abbott Labs as a non-mandated post-market study on 9 March 2016. Funding for this study was provided by Abbott Labs.

### Sample size

A total of 170 subjects at up to 15 investigative sites were planned to be enrolled over ∼18 months.

Since the primary objective of this study relates to the long-term effectiveness of FIRM-guided ablation vs. conventional ablation for AF, power and sample size estimates were calculated relative to that endpoint only. The plan was to enrol 77 subjects in each treatment arm, plus an additional eight subjects per arm to account for possible drop-outs/lost to follow-ups, for a total of 170 subjects (at ∼20 sites). With 77 subjects in each group, the lower limit of the observed one-sided 95.0% confidence interval was expected to exceed −0.150 with 80% power when the standard proportion, pC, is 0.500 and the test expected proportion, pF, is 0.550; results were based on 1000 simulations using the Newcombe–Wilson score method to construct the confidence interval. In order to account for potential drop-outs and losses to follow-up, an additional 10% (16) was added to the total sample size, for a final sample size of 170 (85 per group).^10^ This power and sample size calculation was performed using NQuery 7.0 on the Microsoft Windows 10 operating system.

The first subject was enrolled on 5 February 2016. The actual study duration was ∼2 years, at which time the study was closed early by the sponsor due to slow enrolment after enrolling 51 patients. At the time of study closure, any pending follow-up visits were waived. Termination letters were sent to the sites on 21 February 2018 indicating an immediate close of the study with no further follow-up visits. The last subject was enrolled on 5 February 2018. The last follow-up visit occurred on 23 February 2018.

### Study objectives and endpoints

The primary objective was to compare the safety and effectiveness of FIRM-guided RF ablation alone against the conventional RF-based PVI for the treatment of paroxysmal AF in subjects without prior AF catheter ablation. The two primary efficacy end points were (i) acute success defined as elimination of identified AF rotors (FIRM group) or isolation of all pulmonary veins (PVI group) and (ii) long-term success rate defined as single-procedure freedom from AF or AT recurrence >30 s from 3 to 12 months after the index AF ablation procedure including a 90-day blanking period. Typical atrial flutter was not considered as AT recurrence. The primary safety endpoints were defined as acute (within 7 days of the procedure) or long-term (12 month) freedom from procedure-related serious adverse events (SAE).

### Data management and follow-up

Abbott employees or representatives monitored the study according to company standard operating procedures at each enrolling site at a minimum of 3-month intervals for the purposes of verifying compliance to the protocol and applicable regulations and verifying case report form data to original entries in source files. A protocol deviation was defined as any event where the clinical investigator or site personnel did not conduct the study according to the protocol.

Abbott contracted with Merge (an IBM company) for development and hosting of the Electronic Data Capture system, eClinicalOS (currently V. 2016.4.2). Merge hosted, maintained, and upgraded system software, and securely reposed all clinical data in a validated system. Merge maintained and provided backup for the FIRMAP AF clinical database and front-end entry system. Analyses and data review were performed by Abbott Clinical Affairs and Biometrics staff.

### Device description

The FIRMap™ Basket Catheter (Abbott, Menlo Park, CA, USA) is a multi-electrode catheter that can capture unipolar electrogram data from either the right or left atria. Using the FIRMap™ Catheter, the RhythmView™ System (Abbott, Menlo Park, CA, USA) processes 64 electrograms from areas throughout the cardiac chamber to identify the anatomic locations of these electrical patterns. The RhythmView™ Workstation (Abbott, Menlo Park, CA, USA) provides software tools for graphical display of patient-specific AF sources.

### Atrial fibrillation ablation procedure

Ablation procedure was performed with patients under deep analgosedation.^11^ Antithrombotic therapy with vitamin-K antagonists was continued throughout the procedure. In patients with direct oral anticoagulants, one dose was withheld one day prior to the procedure and restarted on the same evening at half the regular dose, and at full dose the following day without additional bridging with therapeutic heparin. All patients underwent transoesophageal echocardiography prior to the procedure to exclude left atrial appendage thrombus. The right and/or left femoral veins were cannulated after local anaesthesia via Seldinger technique. In the FIRM group, three sheaths were placed in the right femoral vein and two in the left femoral vein and in the conventional group three sheaths were placed in the right femoral vein. A reference catheter was placed into the coronary sinus (CS) and double trans-septal puncture was performed. Two 8.5 French SL1 sheaths (Abbott, St. Paul, MN, USA) were advanced into the left atrium (LA) and an 8.5 French SL1 sheath (Abbott, St. Paul, MN, USA) was advanced into the right atrium (RA) in the FIRM group only. Left atrial sheaths were continuously flushed with heparinized saline and intra-venous heparin was administered to achieve an activated clotting time >300 s. Three-dimensional (3D) electroanatomic maps were created using either the CARTO™ (49/51) (Biosense Webster, Diamond Bar, CA, USA), EnSite NavX™ (1/51) (Abbott, Menlo Park, CA, USA), or Rhythmia™ (1/51) (Boston Scientific, Cambridge, MA, USA) mapping system for documentation of anatomy and ablation lesion locations.

In the conventional PVI group, ablation was performed with the use of an irrigated RF ablation catheter and consisted of isolation of all four PVs with confirmed entrance block assessed by a multipolar catheter.

**Figure 1:**
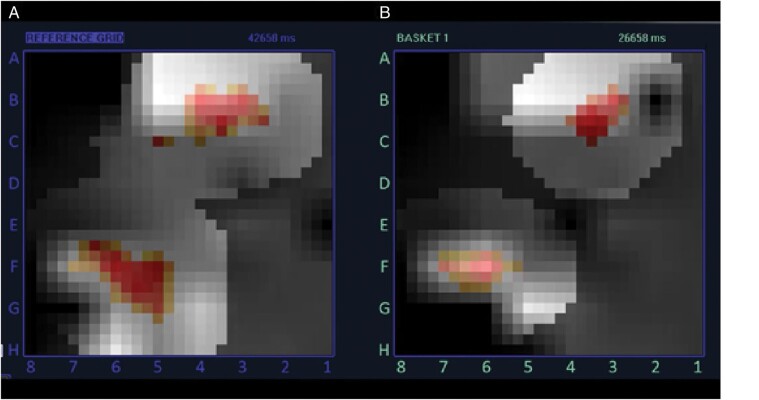
Isochronal activation map of two rotors reconstructed from data obtained using the 64-electrode FIRMap™ basket catheter and RhythmView™ computational mapping system. The rotors could be reproducibly located at the same location in two consecutive maps (*A* ‘Reference’, *B*).

In the FIRM group rotor mapping was performed as previously described.^12^ In brief, if the patient did not experience spontaneous AF, sustained AF (>5 min uninterrupted) was induced by atrial burst pacing with or without isoproterenol infusion. A 64-electrode FIRMap™ basket catheter was introduced into the RA, followed by the LA. Unipolar and bipolar electrograms from the basket catheter were filtered at 0.05–500 Hz, recorded at a 1 kHz sampling frequency with a notch filter of 50 Hz using a commercially available electrophysiological recording system and exported to RhythmView™, the computational FIRM mapping system that generates an AF propagation map (*Figure 1*). Each one-minute recording was termed an ‘Epoch’. A four second time segment from each Epoch was analysed to create a rotor map. The ‘spotlight^TM’^ and ‘RAP^TM^’ tools were used to standardize identification of rotors. To demonstrate rotor stability, rotor mapping was repeated at each position and only rotors that were confirmed by a second, independent map were targeted for ablation. First right atrial mapping was performed. If no right atrial rotor/source was detected or all right atrial rotors were eliminated, left atrial mapping was performed. Identified rotors or focal impulses were transferred and marked in the 3D mapping system. Then, RF ablation in the rotor core region was performed for at least 300 s covering the rotor core and the immediate surrounding area (∼2 cm^2^).^13^ Afterwards, repeat mapping was performed to confirm that the rotor had been eliminated. Following each FIRM-guided site ablation, FIRM mapping was repeated and followed by rotor ablation until all rotors were eliminated or AF terminated and remained non-inducible. Power was limited to 20–25 W at the posterior wall and 35–40 W in the remaining left and RA.

### Post-procedure drug therapy

Post-procedure drug therapy included re-initiation and/or continuation of any pre-procedure cardiac medications. Anti-arrhythmic drugs (AAD) were initiated or continued 3 months post-ablation. Afterwards, AAD were encouraged to be discontinued but were left at the discretion of the treating physician.

Oral anticoagulants were continued for at least 3 months post-ablation and thereafter dependent on the CHA_2_DS_2_-VASc score.

### Rhythm follow-up

Post-procedure follow-up included inpatient visits at 3, 6, and 12 months with 12-lead ECG, 72 h ambulatory continuous ECG monitor (Holter), assessment of symptom and rhythm, EHRA class, concomitant anti-arrhythmic medications, and adverse events. Analysis of the Holter readings was performed by the medical monitor, who was blinded to study treatment. Arrhythmia recurrence was defined as documented episodes of AF/AT >30 s.

### Statistical analysis

All primary endpoint analyses were conducted under the principle of ‘Intention-To-Treat’ (ITT). In the study protocol, the ITT group was defined as all subjects randomized to a treatment group who had a mapping and/or ablation catheter inserted.

The primary effectiveness endpoint was defined as freedom from AF recurrence during 3–12-month follow-up period post-index AF catheter ablation procedure. This study was designed to test the hypothesis that the recurrence rate for FIRM-guided AF ablation is not inferior to that experienced by the conventional PVI arm. The proportion of success in each treatment arm was estimated using Kaplan–Meier survival estimation. Due to the early closure of the study, there were insufficient patient numbers to perform a powered test of this hypothesis. However, log rank comparison of time with arrhythmia recurrences was performed.

Continuous variables are presented as mean ± standard deviation (SD). For highly skewed variables, median, and interquartile range (IQR) are given. Categorical variables are expressed as number and percentage of patients. The statistics shown should be regarded as descriptive and were based on the available cases.

All analyses were performed by the sponsor using SAS.

## Results

### Patient characteristics

**Figure 2:**
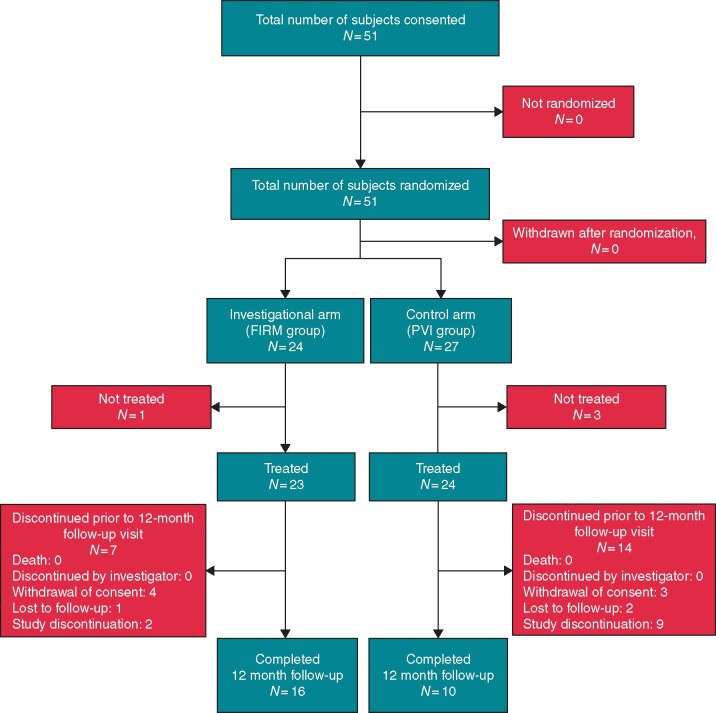
Study flow.

From February 2016 until February 2018 a total of 51 patients with paroxysmal AF, were consented and enrolled at two sites (UKSH Luebeck 47 patients, Unfallkrankenhaus Berlin 4 patients, *Figure 2*): Four patients withdrew from the study prior to treatment. A total of 47 patients were treated: 23 in the investigational FIRM group and 24 in the control PVI group. Patient characteristics were similar between both groups with mean patient age 63 ± 10.6 years, a slight majority (56.9%) being male and the majority of patients presenting in EHRA class II and III. Additional demographic details are shown in *Table 1*.

**Table 1:** Demographics and baseline characteristics

	FIRMap (*N* = 24)	Conventional (*N* = 27)	All subjects (*N* = 51)
Age (years), mean ± SD (*n*), median [IQR]	62 ± 11.4 (24), 65 [33–78]	65 ± 9.7 (27), 68 [48–78]	63 ± 10.6 (51), 65 [33–78]
Sex, male	14/24 (58.3%)	15/27 (55.6%)	29/51 (56.9%)
BMI (kg/m^2^), mean ± SD (*n*), median [IQR]	27 ± 6.2 (24), 25 [18–39]	28 ± 5.5 (26), 27 [19–43]	28 ± 5.8 (50), 26 [18–43]
EHRA class			
^ ^Class I	2/24 (8.3%)	0/27 (0.0%)	2/51 (3.9%)
^ ^Class II	8/24 (33.3%)	7/27 (25.9%)	15/51 (29.4%)
^ ^Class III	14/24 (58.3%)	18/27 (66.7%)	32/51 (62.7%)
^ ^Class IV	0/24 (0.0%)	1/27 (3.7%)	1/51 (2.0%)
^ ^Unknown	0/24 (0.0%)	1/27 (3.7%)	1/51 (2.0%)
Medical history			
^ ^Arterial hypertension	11/20 (55.0%)	13/21 (61.9%)	24/41 (58.5%)
^ ^Coronary artery disease	8/20 (40.0%)	8/21 (38.1%)	16/41 (39.0%)
^ ^Chronic renal insufficiency	2/20 (10.0%)	3/21 (14.3%)	5/41 (12.2%)
Anti-arrhythmic use at baseline (%)			
^ ^Amiodarone	2/24 (8.3%)	0/25 (0.0%)	2/49 (4.1%)
^ ^Dronedarone	0/24 (0.0%)	0/25 (0.0%)	0/49 (0.0%)
^ ^Flecainide	4/24 (16.7%)	7/25 (28.0%)	11/49 (22.4%)
^ ^Propafenone	1/24 (4.2%)	0/25 (0.0%)	1/49 (2.0%)
^ ^Sotalol	0/24 (0.0%)	2/25 (8.0%)	2/49 (4.1%)
Left atrial diameter (mm); mean ± SD (*n*), median [IQR]	44 ± 5.5 (24), 43 [36–55]	44 ± 7.3 (23), 42 [32–67]	44 ± 6.4 (47), 42 [32–67]

IQR, interquartile range.

### Procedural data

Total procedure time in the FIRM group was 156 ± 60 min, compared with 104 ± 58 min in the PVI group (*Table 2*). This difference was primarily due to differences in the RF ablation time (117 ± 37 min in FIRM group vs. 77 ± 41 min in PVI group), which was defined as the period from first to last ablation. In the FIRM group, this period included the additional mapping time required for identification of rotors and confirmation of rotor elimination. In the PVI group, mapping was conducted prior to ablation and therefore not included in this period.

**Table 2:** Procedural data

	FIRMap (N = 24)	Conventional (N = 27)	All subjects (N = 51)
Total procedure time (min); mean ± SD (*n*), median [IQR]	156 ± 59.8 (21), 155 [2–280]	104 ± 58.1 (24), 100 [8–247]	129 ± 63.9 (45), 120 [2–280]
Total RF ablation time^a^ (min); mean ± SD (*n*), median [IQR]	117 ± 37.1 (21), 120 [70–189]	77 ± 40.9 (23), 60 [10–175]	96 ± 43.8 (44), 87 [10–189]
Total fluoroscopy time (min); mean ± SD (*n*), median [IQR]	16 ± 19.6 (22), 12 [5–101]	14 ± 10.9 (23), 10 [0–52]	15 ± 15.6 (45), 11 [0–101]

^a^Due to low number of patients statistical analysis of this parameter was not performed and rather presented as descriptive data. IQR, interquartile range; RF, radiofrequency.

### Rotor identification

**Figure 3:**
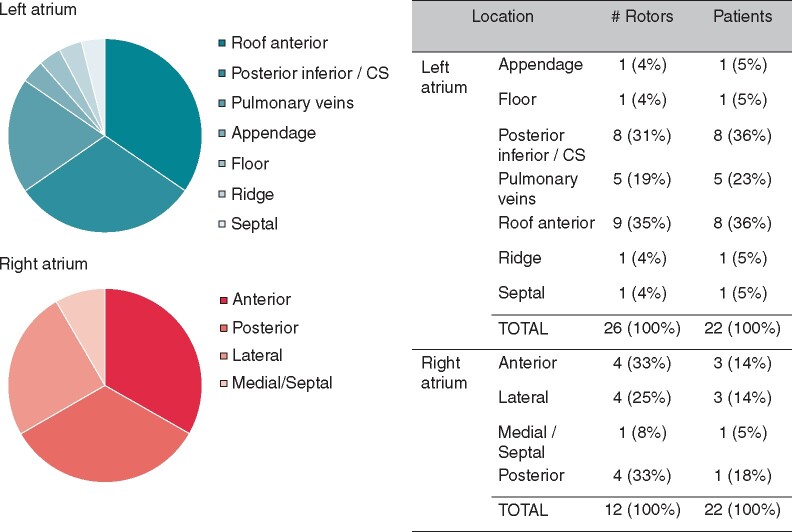
Location of identified rotors. Rotors were identified and localized in FIRM group patients (*n* = 22). Distribution of rotor location is shown for the left (*A*) and right (*B*) atrium. (*C*) Details on the number of rotors and the proportion of patients with at least one rotor in each location.

Rotor localization information was collected from 22 patients in the FIRM group. Two-thirds of identified rotors were in the LA (26 out of 38, 68%). The locations of identified rotors are summarized in *Figure 3*. In the LA, most rotors were in the roof (anterior) and posterior inferior near the CS (9 and 8 out of 26, respectively). Each individual patient had up to three rotors identified and ablated (mean 1.6 ± 0.15, median 1.5).

### Primary endpoints

#### Elimination of rotors and pulmonary vein isolation

The acute primary efficacy endpoint defined as elimination of all identified AF rotors in the FIRM group or isolation of all PVs in the PVI group was achieved in all treated patients (24/24 in FIRM group and 27/27 in PVI group).

#### Freedom from atrial fibrillation/at recurrence

Median follow-up time for the total number of randomized patients was 366.0 (355.0–373.0) days in the FIRM group and 370.5 (365.0–374.0) in the conventional group.

**Figure 4:**
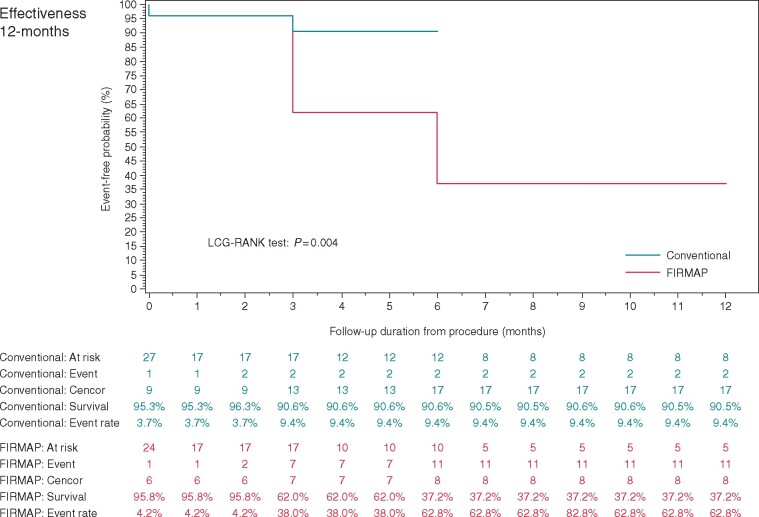
Kaplan–Meier analyses for single-procedure effectiveness at 12-month follow-up.

With respect to patients treated as randomized single-procedure effectiveness (freedom from AF/AT recurrence after blanking period) was 52.9% (9/17) in the FIRM group and 85.7% (12/14) in the PVI group at 6 months [0.327 (confidence interval −0.003 to 0.57); log-rank *P*-value 0.035]; and 31.3% (5/16) in the FIRM group and 80% (8/10) in the PVI group at 12 months [0.488 (confidence interval 0.09–0.71); log-rank *P*-value 0.004] (*Figure 4*).

Repeat procedures were performed in 45.8% (11/24) patients in the FIRM group and 7.4% (2/27) in the PVI group. None of the repeat procedures were reported as FIRM guided. Of the 11 FIRM subjects that had repeat procedures, 3 were free from recurrence at 12 months and the remaining 9 did not have 12-month data available. Repeat hospitalizations any time after initial discharge (including the 3 months ‘blanking’ period) occurred in 7/24 (29.2%) patients in the FIRM group and 4/27 (14.8%) in the PVI group, respectively (*Table 3*).

**Table 3:** Long-term freedom from AF/AT recurrence

	FIRMap (*N* = 24)	Conventional (*N* = 27)	All subjects (*N* = 51)
Subjects with repeat procedure(s)^a^	11/24 (45.8%)	2/27 (7.4%)	13/51 (25.5%)
Subjects with repeat hospitalization(s)^a^	7/24 (29.2%)	4/27 (14.8%)	11/51 (21.6%)
Single-procedure freedom from AF/AT recurrence at 6 months post-index ablation procedure (with 90-day blanking period)	9/17 (52.9%)	12/14 (85.7%)	21/31 (67.7%)
Single-procedure freedom from AF/AT recurrence at 12 months post-index ablation procedure (with 90-day blanking period)	5/16 (31.3%)	8/10 (80.0%)	13/26 (50.0%)

AF, atrial fibrillation; AT, atrial tachycardia.

a Repeat procedures or hospitalizations any time after initial discharge (including the time during the 3 months ‘blanking’ period).

#### Safety endpoints

The acute safety endpoint (freedom from procedure-related SAEs) was achieved in 87% (20/23) of FIRM group patients and 100% (24/24) of PVI group patients. Procedure-related SAEs occurred in three patients in the FIRM group: one femoral artery aneurysm and two injection site haematomas. There were no device-related SAEs. No additional procedure-related SAEs were reported >7 days post-procedure. There were no deaths reported in this study.

## Discussion

### Main findings of the study

To the best of our knowledge, the FIRMAP AF trial was the first randomized trial assessing the safety and efficacy of rotor ablation as the sole ablative strategy (without PVI) compared with the gold standard of conventional PVI in patients with paroxysmal AF. We found that (i) Elimination of all rotors in the FIRM group and isolation of all pulmonary veins in the PVI group could be achieved in all patients. (ii) Single-procedure effectiveness was very poor in the FIRM group as compared with the PVI group at 6 months (52.9% vs. 85.7%) and 12 months (31.3% vs. 80%), respectively. (iii) Repeat catheter ablation procedures were performed more frequently in the FIRM group than in the PVI group (45.8% vs. 7.4%). (iv) Complication rates were low in both arms, with three vascular access site complications in the FIRM group and no major SAEs or device-related events in the PVI arm.

A substantial number of patients experience recurrence of AF after PVI, which currently presents the gold standard for AF catheter ablation. This has led to a search for further underlying mechanisms for AF maintenance. In this light ablation of rapidly spinning rotors or very rapid focal impulse formation has been shown to increase success rates after AF catheter ablation.^3–6^^,^^8^ Recent studies even highlight these rotors and impulse formations as the crucial mechanism of AF maintenance.^1^^,^^8^ This is supported by analyses demonstrating that PVI rather presents a coincidental ablation of rotors.^1^^,^^8^ Furthermore, the PRECISE trial indicated that FIRM ablation only is associated with high single procedure AF ablation success rates of 82.6% in paroxysmal AF patients.^9^ Earlier results of our group could reproduce a significant influence of rotor ablation on AF slowing or termination, while success rate after 13 months was lower (52%) in this single centre non-randomized study that mostly included non-paroxysmal AF patients (60%).^12^

Several reviews and meta-analyses conclude that AF driver-guided catheter ablation may increase freedom from AF/AT relative to conventional strategies, while highlighting the fact that these conclusions are based on non-randomized studies of moderate quality.^3–6^ Based on these data and on the previous hypothesis that PVI presents a coincidental ablation of rotors, we aimed to assess the impact of FIRM-guided ablation compared with the gold standard of PVI in a randomized fashion in patients with paroxysmal AF.

It was shown that using the FIRMap™ catheter and the RhythmView™ system, a mean of 1.6 ± 0.15 rotors could be identified in the right (*n* = 12) and left (*n* = 26) atrium. This is in line with previous studies indicating that most sources can be located in the LA.^8^^,^^12^ Successful ablation of these rotors was demonstrated in all patients.

In our study, single-procedure effectiveness at 6 and 12 months was lower in the FIRM-guided ablation arm as compared with the PVI arm: 52.9% vs. 85.7% at 6 months and 31.3% vs. 80.0% at 12 months, respectively. These results suggest that PVI should still present the cornerstone of AF ablation in patients with paroxysmal AF, instead of presenting a coincidental ablation effect. Reasons for worse outcome on FIRM-guided patients may relate to a potentially arrhythmogenic effect of rotor ablation alone. To further address this point assessment of mode of arrhythmia recurrence would be of interest. This has not been systematically evaluated in this study, but will be addressed within the prospective Lübecker ablation registry.

The value of FIRM ablation in non-paroxysmal AF patients still has to be elucidated. The Outcome of Different Ablation Strategies In Persistent and Long-Standing Persistent Atrial Fibrillation (OASIS) trial (NCT02533843) evaluated the impact of FIRM ablation with or without PVI vs. PVI plus non-PV trigger ablation in patients with persistent and long-standing persistent AF. However, this publication has been retracted for randomization issues. Nevertheless, it should be noted that enrolment in the FIRM only ablation group in the OASIS trial was terminated early after an unplanned interim assessment by the internal safety committee due to a high AF recurrence rate.

The only other randomized data on FIRM ablation in patients with persistent AF have recently been presented at the Heart Rhythm Congress 2019.^14^ The Randomized Evaluation of Atrial Fibrillation Treatment with Focal Impulse and Rotor Modulation-guided procedures (REAFFIRM) trial is a prospective multicentre, randomized, controlled trial comparing conventional PVI with FIRM-guided ablation in addition to PVI in persistent AF.^14^ First results indicate that single procedure freedom from AF/AT recurrence as well as the percentage of repeat procedures did not differ significantly between FIRM-guided and conventional therapy.^14^ Publication of results is still pending.

The Redo-FIRM study (ClinicalTrials.gov Identifier: NCT02799043), which assesses the impact of FIRM-guided ablation in addition to redo-PVI vs. redo-PVI only in patients with recurrence of paroxysmal or persistent AF, is currently still ongoing.

### Complications

Complication rate was low in this study, with three vascular access site complications occurring in the FIRM group vs. none in the conventional group. The small patient population for the study precludes the conclusion that this may relate to the higher number of vascular access sheaths needed in the FIRM group (three sheaths to the right femoral vein and two to the left femoral vein vs. three sheaths only in the conventional arm). There were no other device or procedure-related SAEs reported.

### Limitations

Limitations of this analysis relate to the small number of patients which precludes statistical testing. At the time this study was initiated, it was unclear whether PVI (elimination of triggers) or rotor ablation (to prevent propagation) would be the superior strategy for treatment of paroxysmal AF. During the course of this study, additional data and guidance were published establishing PVI as the cornerstone of AF treatment. This updated guidance and additional published data may have contributed to a drop-off in enrolments and subsequent study closure. Due to the early study closure, 6 and 12 month follow-up data are limited to 24 and 27 patients only, respectively. However, randomized study design and paucity of data strengthen the importance of publishing this study. Furthermore, patients undergoing ablation at the University Hospital in Lübeck were also enrolled in the Lübecker ablation registry. Publication of longer-term follow-up data of these FIRM ablated paroxysmal AF patients compared with patients ablated with the cryoballoon are expected soon.

## Conclusion

These study results reinforce the importance of PVI in paroxysmal AF patients and suggest that FIRM-guided ablation alone (without PVI) is not an effective strategy for treatment of paroxysmal AF in most patients. Further study is needed to understand the effectiveness of adding FIRM-guided ablation as an adjunct to PVI in this patient group.

## Funding

Funding for this study was provided by Abbott, Topera Inc., Menlo Park, CA 94025, USA. Contract number: 20160108. 

**Conflict of interest:** R.R.T. received research grants from Medtronic, Biotronik, travel grants from Biosense Webster, Medtronic, Abbott, SentreHeart, and Daiichi Sankyo and speaker’s bureau/proctor honoraria from Biosense Webster, Medtronic, Abbott, Sentrheart, and Daiichi Sankyo; he is consultant of Biosense Webster and Biotronik. C.L. is consultant of Biosense Webster. P.S. received modest lecture fees from Abbott and is part of the advisory board of Abbott. C.H.H. received travel grants and research grants by Medtronic, Claret Medical, SentreHeart, Biosense Webster, and Cardiofocus. He received speaker’s bureau/proctor honoraria from Cardiofocus and Medtronic. S.S. and A.S. are employees of Abbott Labs. J.V. received travel grants from Bayer, Biosense Webster, and Daiichi Sankyo and speaker’s honoraria from Abott, Daiichi Sankyo, and Novartis. C.E. received travel grants from Biosense Webster, Medtronic, Biotronik, Abbot, and Daiichi Sankyo and speaker’s honoraria from Biosense Webster, Medtronic, Abbott, Sentrheart, and Daiichi Sankyo. The other authors report no conflicts of interest.

## Data availability

The data underlying this article will be shared on reasonable request to the corresponding author.
